# Quantitative accuracy of virtual non-contrast images derived from spectral detector computed tomography: an abdominal phantom study

**DOI:** 10.1038/s41598-020-78518-5

**Published:** 2020-12-09

**Authors:** Jasmin A. Holz, Hatem Alkadhi, Kai R. Laukamp, Simon Lennartz, Carola Heneweer, Michael Püsken, Thorsten Persigehl, David Maintz, Nils Große Hokamp

**Affiliations:** 1 Faculty of Medicine and University Hospital Cologne, Institute for Diagnostic and Interventional Radiology, University of Cologne, Kerpener Strasse 62, 50937 Cologne, Germany; 2 Department of Radiation Oncology, University Hospital Bonn, University of Bonn, Venusberg Campus 1, 53127 Bonn, Germany; 3 Institute of Diagnostic and Interventional Radiology, University Hospital Zurich, University of Zurich, Rämistrasse 100, 8091 Zurich, CH Switzerland

**Keywords:** Medical imaging, Tomography

## Abstract

Dual-energy CT allows for the reconstruction of virtual non-contrast (VNC) images. VNC images have the potential to replace true non-contrast scans in various clinical applications. This study investigated the quantitative accuracy of VNC attenuation images considering different parameters for acquisition and reconstruction. An abdomen phantom with 7 different tissue types (different combinations of 3 base materials and 5 iodine concentrations) was scanned using a spectral detector CT (SDCT). Different phantom sizes (S, M, L), volume computed tomography dose indices (CTDIvol 10, 15, 20 mGy), kernel settings (soft, standard, sharp), and denoising levels (low, medium, high) were tested. Conventional and VNC images were reconstructed and analyzed based on regions of interest (ROI). Mean and standard deviation were recorded and differences in attenuation between corresponding base materials and VNC was calculated (VNCerror). Statistic analysis included ANOVA, Wilcoxon test and multivariate regression analysis. Overall, the VNC_error_ was − 1.4 ± 6.1 HU. While radiation dose, kernel setting, and denoising level did not influence VNC_error_ significantly, phantom size, iodine content and base material had a significant effect (e.g. S vs. M: − 1.2 ± 4.9 HU vs. − 2.1 ± 6.0 HU; 0.0 mg/ml vs. 5.0 mg/ml: − 4.0 ± 3.5 HU vs. 5.1 ± 5.0 HU and 35-HU-base vs. 54-HU-base: − 3.5 ± 4.4 HU vs. 0.7 ± 6.5; all *p* ≤ 0.05). The overall accuracy of VNC images from SDCT is high and independent from dose, kernel, and denoising settings; however, shows a dependency on patient size, base material, and iodine content; particularly the latter results in small, yet, noticeable differences in VNC attenuation.

## Introduction

Computed tomography (CT) provides morphological images with a high spatial resolution. To overcome the intrinsic low soft tissue contrast of CT, iodinated contrast media is frequently administered, particularly in abdominal CT. Modern dual-energy CT’s (DECT) allow for the reconstruction of virtual non-contrast (VNC) images, which have the potential to replace true non-contrast scans.

Conventional CT uses one polychromatic x-ray source emitting a wide energy spectrum, typically covering a range from 40–120 (− 140) kVp. In conventional CT, a scintillation detector registers the global loss of intensity due to tissue attenuation. Hence, a global attenuation profile of the scanned object is obtained. Images reconstructed from this data are visualized using Hounsfield units (HU). As such a global attenuation profile in single energy CT cannot differentiate the influence of Photoelectric effect and Compton scattering, two different materials may exhibit similar HU values. To overcome this limitation of conventional CT, dual-energy CT can be used. In DECT, two different energy spectra are employed to obtain separate attenuation profiles for lower and higher energy photons. Technological approaches to DECT are either emission-based (dual-source CT (DSCT), fast kV_p_-switching DECT or twin-beam DECT) or detector-based (dual-layer, spectral detector CT (SDCT))^1–3^.

By separate assessment of low and high energy attenuation profiles, material decomposition becomes possible^4–6^. The most abundant applications of material decomposition comprise material specific maps for gout and iodine. In the latter, distribution of iodinated contrast media is visualized in quantitative maps^4,7–11^. These allow for the reconstruction of images that subtract the iodine-associated attenuation resulting in so-called virtual non-contrast (VNC) images^12–20^. While several studies demonstrated the general functionality of VNC^21–26^; studies which systematically investigating the impact of different patient-associated sizes, image acquisition and reconstruction parameters are sparse. Yet, it is essential to understand confounding factors for the evaluation of VNC performance^8–11,27–30^.

This study aims to comprehensively evaluate these parameters using an anthropomorphic abdomen phantom.

## Methods

### Phantom description

**Figure 1:**
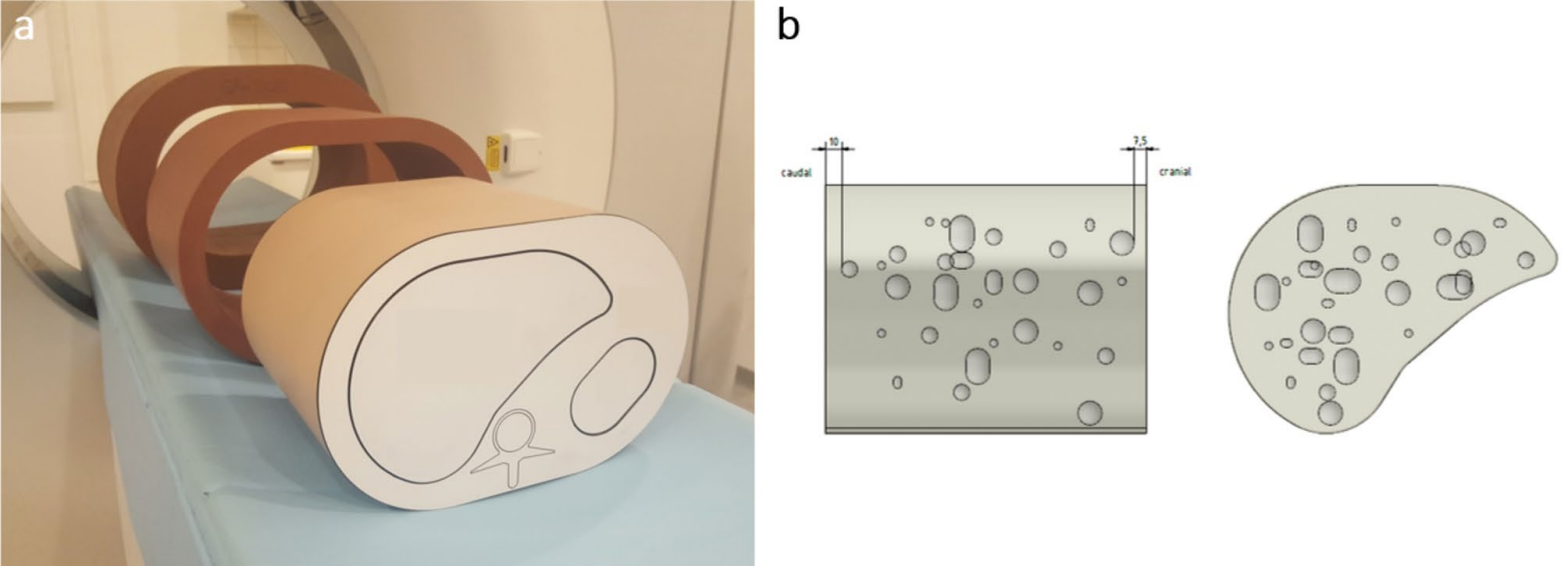
Anthropomorphic abdomen phantom with liver insert. (**a**) Photo of abdomen phantom size S and extension rings (employed to simulate sizes M and L). (**b**) Scheme of liver insert with lesion location and size in axial and sagittal plane.

An anthropomorphic abdomen phantom with liver insert was used (QSA-453 and QSA-637, QRM GmbH, Moehrendorf, Germany) containing a total of 17 liver lesions with a size of > 5 mm (8 hyperdense and 9 hypodense lesions; Fig. 1).

Different patient sizes were simulated using no extension ring small (S, 300 × 200 mm), and with extension rings in size medium (M, 350 × 250 mm) and large (L, 400 × 300 mm). Matrix material of the phantom exhibited an attenuation of 35 HU on conventional CT without iodine present. According to the vendor, the parenchyma’s base material yields attenuation of 54 HU, which is increased by adding iodine to meet attenuation of 90 HU on conventional images (Table 1). Base material and iodine amount of liver lesions varied to meet clinically encountered attenuation values of 45, 60, 120 and 180 HU on conventional images (Table 1, Electronic supplement 1). In total, this resulted in 7 different types of tissue (5 lesion types + matrix + parenchyma, Table 1). Of note, iodine concentrations provided by the vendor were established using a DSCT; therefore, no accuracy calculations of iodine were conducted.

**Table 1:** Specification of tissue types with liver lesions and size > 5 mm. Of note, iodine concentrations as provided by the vendor are estimates only (as per phantom datasheet).

Tissue type	Total attenuation (HU)	Base attenuation (HU)	Iodine (mg/ml)	10 (mm)	15 (mm)	10 × 15 (mm)	15 × 22.5 (mm)
Matrix	35	35	0.0	–	–	–	–
Parenchyma	90	54	1.4	–	–	–	–
Lesion type 1	45	45	0.0	1	2	–	1
Lesion type 2	60	54	0.3	1	–	–	1
Lesion type 3	45	35	0.4	1	–	1	1
Lesion type 4	120	54	3.0	1	1	1	1
Lesion type 5	180	54	5.0	1	1	1	1

### Data acquisition and image reconstruction

The phantom was placed in the isocenter of a spectral detector CT (SDCT, IQon, Philips Healthcare, Best, The Netherlands) and scanned with a slightly modified routine clinical abdomen protocol: tube voltage 120 kVp, fixed tube current–time product of 111 mAs, 166 mAs, 222 mAs, resulting in volume computed tomography dose index CTDI_vol_ of 10 mGy, 15 mGy and 20 mGy; field-of-view of 350 mm, 400 mm and 450 mm for phantom size S, M and L, respectively; collimation 64 × 0.625 mm; pitch 0.485; rotation time 0.33 s; matrix 512 × 512.

Conventional images were reconstructed using the vendor’s hybrid-iterative reconstruction algorithm (iDose^4^, Philips Healthcare, Best, The Netherlands). Virtual non-contrast images and iodine maps were reconstructed using a dedicated spectral reconstruction algorithm (Spectral, Philips Healthcare, Best, The Netherlands). As the reconstruction algorithm involves methods from iterative reconstruction, it allows for a choice of denoising level; here, no, medium, and strong denoising levels were chosen (0/6, 3/6 and 6/6, respectively)^31^. Furthermore, image definition (kernel) was varied between soft, standard, and sharp (A, B and C, Philips Healthcare, Best, The Netherlands). All images were reconstructed with a slice thickness of 2 mm with a section increment of 1 mm in axial plane (analogously to our institution’s routine protocol). In total, the variation resulted in 81 reconstructions (3 patient sizes × 3 radiation doses × 3 denoising settings × 3 kernels).

### Data collection

Data was collected based on region of interest measurement (ROI). ROI were placed on conventional images, copied, and pasted to iodine maps and VNC images, respectively. ROI were placed in all 17 lesions (one ROI each), in the parenchyma (i.e., spleen insert) as well as the matrix material (two ROI each) and drawn as large as possible (d ≥ 6 mm). Attenuation on conventional and VNC images, as well as iodine concentration on iodine maps were recorded including corresponding standard deviations.

The VNC_error_ was calculated as difference between attenuation in VNC reconstruction (HU_VNC_) and the reported attenaution of the corresponding base material (HU_base_):

$${VNC}_{error}={HU}_{VNC}-{HU}_{base}$$

Lesions with none, low, medium, and high iodine concentrations were grouped as indicated (0.0 mg/ml–0.3 and 0.4 mg/ml–3.0 and 5.0 mg/ml, respectively).

### Data analysis

All statistical analysis was performed using JMP Software (SAS Institute, Gary, USA). Continuous data is presented as mean ± standard deviation (SD). Statistical analysis was carried out using one-way ANOVA or Wilcoxon signed-rank test with Tukey–Kramer and Steel–Dwass adjustment for multiple comparisons, respectively. Further multivariate regression analyis was used. Statistical significance was defined as *p* ≤ 0.05. Waterfall diagrams were used to visualize VNC_error_ and its dependence on patient size, base material, and iodine content.

## Results

### Attenuation characteristics

**Figure 2:**
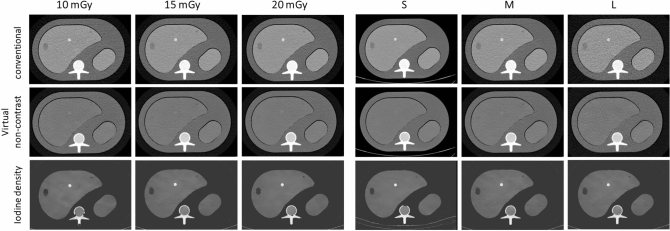
Example of axial slice images of liver lesions. Conventional (upper row), virtual non-contrast (middle row), and iodine density (lower row) images: LEFT) images of the medium sized phantom acquired with a CTDIvol of 10, 15, and 20 mGy are shown. RIGHT) images in different sizes (small, medium and large) are shown. Of note, conventional and VNC images are displayed with the institutional defaults for window level and width (WL 60, WW 360) and iodine (WL 2, WW 7), respectively. Images are cropped, hence the extension ring is not visible in S- and L-phantoms.

Overall, attenuation ranged from 24.9–195.6 HU and 18.7–72.5 HU in conventional and VNC images, respectively. Iodine concentration ranged from 0.0 to 5.5 mg/ml. Average VNC_error_ was − 1.4 ± 6.1 HU, ranging from − 16.3 to 18.5 HU. Detailed values for pooled analysis of all measurements are depicted in Table 2, example images are shown in Fig. 2.

**Table 2:** Overall measurement results for attenuation, VNC, iodine and VNCerror for each tissue type.

Tissue type	Attenuation (HU)		VNC (HU)		Iodine (mg/ml)		VNC_error_ (HU)	
	Mean	SD	Mean	SD	Mean	SD	Mean	SD
Matrix	30.3	2.4	33.0	1.1	0.0	0.0	− 2.0	1.1
Parenchyma	96.5	1.5	58.9	0.8	1.4	0.1	4.9	0.8
Lesion type 1	46.6	3.2	40.0	3.9	0.2	0.1	− 5.0	3.9
Lesion type 2	58.7	3.8	47.8	4.0	0.4	0.2	− 6.2	4.0
Lesion type 3	43.8	4.8	30.5	5.4	0.5	0.2	− 4.5	5.4
Lesion type 4	117.3	4.9	51.6	5.5	2.6	0.3	− 2.4	5.5
Lesion type 5	181.8	5.8	59.1	5.0	4.8	0.3	5.1	5.0

### VNC error and phantom size

**Figure 3:**
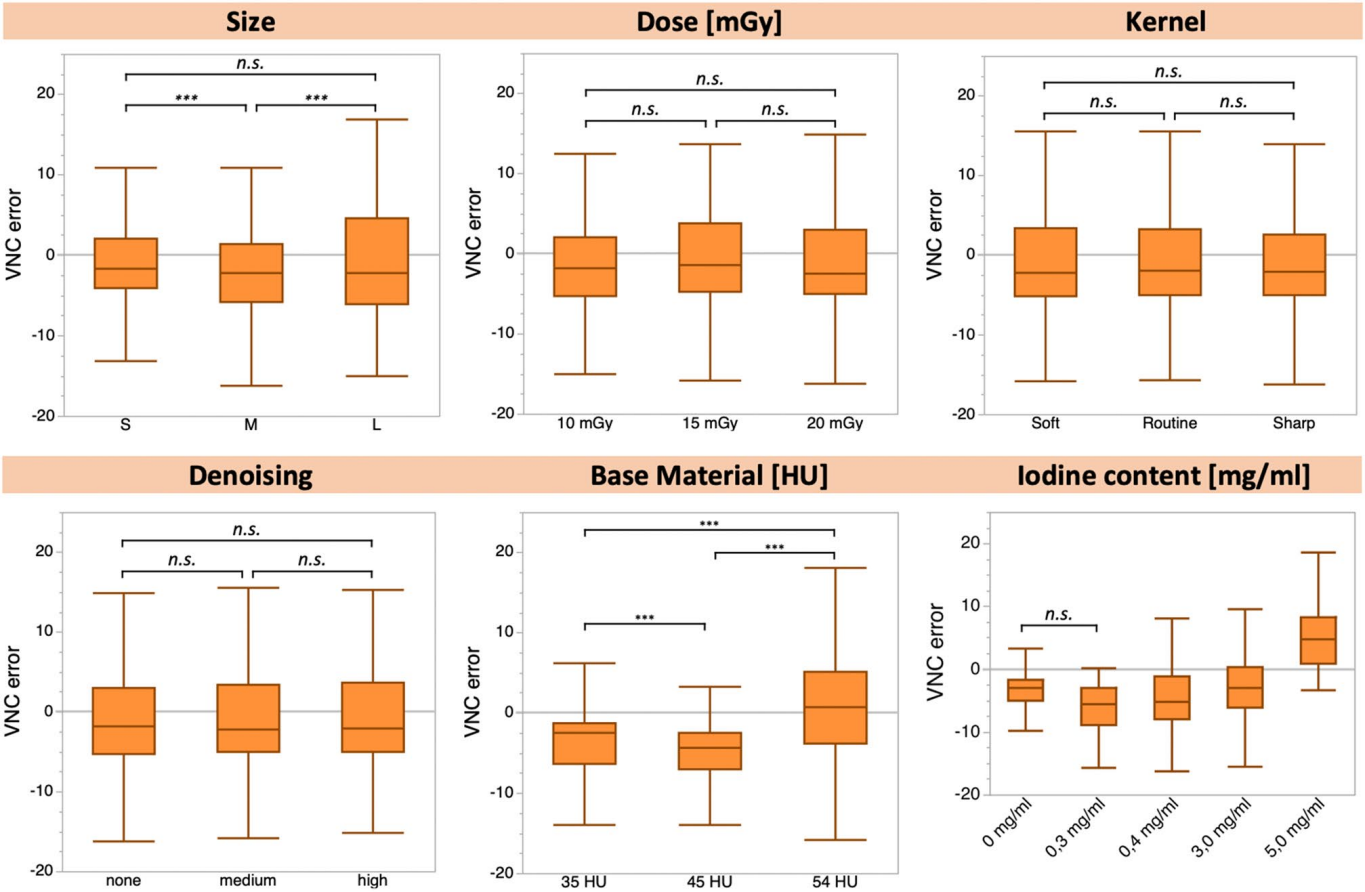
Box plots of the median VNC_error_ for phantom sizes, radiation doses, kernel settings, denoising levels, base materials, and iodine contents. Significant differences are indicated (***p < 0.001, n.s. *p* > 0.05), except for iodine content (bottom right), for which all groups exhibit statistically significant (*p* ≤ 0.05) differences (unless indicated).

No significant differences were found between small and large phantom settings (VNC_error_: − 1.2 ± 4.9 HU and − 0.9 ± 7.1 HU, respectively, *p* ≥ 0.05, Fig. 3). Between small and medium as well as medium and large phantom settings, significant differences in VNC_error_ were observed (VNC_error_: − 1.2 ± 4.9 HU, − 2.1 ± 6.0 HU and − 0.9 ± 7.1 HU, respectively, *p* ≤ 0.05, Fig. 3).

### VNC error and radiation dose

No significant differences were found between 10 mGy, 15 mGy and 20 mGy acquisitions (VNC_error_: − 1.2 ± 6.5 HU, − 1.2 ± 5.7 HU and − 1.7 ± 6.1 HU, respectively, *p* ≥ 0.05, Fig. 3).

### VNC error and image definition (kernel)

No significant differences were found between soft, standard or sharp image definition (kernel), (VNC_error_: − 1.4 ± 6.1 HU for all kernels, *p* ≥ 0.05, Fig. 3).

### VNC error and denoising preset

No significant differences were found between no, medium and strong denoising, (VNC_error_: − 1.4 ± 6.1 HU for all denoising levels, *p* ≥ 0.05, Fig. 3).

### VNC error and base material

Between all three base materials, significant differences were found (VNC_error_: 35-HU, 45-HU and 54-HU-base: − 3.5 ± 4.4 HU, − 5.0 ± 3.9 HU and 0.7 ± 6.5 HU, respectively, *p* ≤ 0.001, Fig. 3).

### VNC error and iodine concentration

Between different iodine contents differences in VNC_error_ were observed (0 mg/ml: − 4.0 ± 3.5 HU; 0.3 mg/ml: − 6.2 ± 4.0 HU; 0.4 mg/ml: − 4.5 ± 5.4 HU; 3.0 mg/ml: − 2.4 ± 5.5 HU; 5.0 mg/ml: 5.1 ± 5.0 HU; 1.4 mg/ml: 4.9 ± 0.8 HU, all *p* ≤ 0.05, except 0.0 mg/ml versus 0.3 mg/ml: *p* > 0.05; Fig. 3).

### Regression analysis

**Figure 4:**
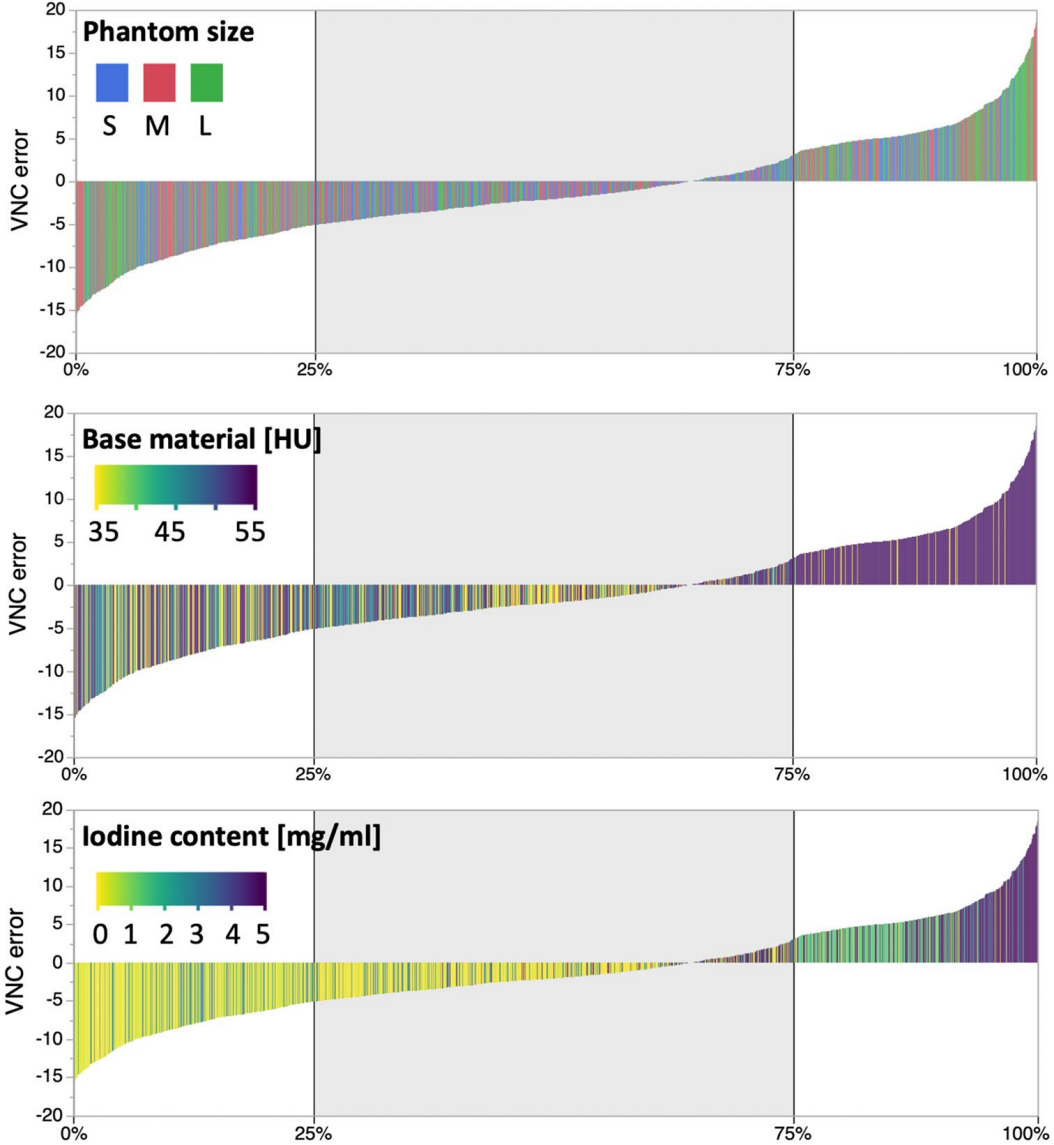
Waterfall diagrams visualize the all VNC_error_ sorted in ascending order and color-coded in dependency of phantom size, base material, and iodine content.

In line with results from inter-group comparison, phantom size, base material, and iodine content were deemed significant parameters of VNC_error_ in regression analysis (all *p* ≤ 0.001). Dose, kernel and denoising level, on the other hand, did not reach significance (*p* = 0.178, *p* = 0.973 and *p* = 0.879). In visual analysis, the impact of phantom size is not depictable in waterfall plots (Fig. 4), while it can be acknowledged that higher attenuation of base material as well as higher iodine content result in a positive VNC_error_.

## Discussion

This study systematically evaluated the influence of size, radiation dose, kernel, denoising level, base material, and iodine content on the accuracy of virtual non-contrast images using an anthropomorphic abdomen phantom. The key findings are that VNC performance is independent of radiation dose, kernel setting and denoising level, while patient size, base material and iodine concentration decrease performance slightly.

Overall, our data suggests a high accuracy of VNC with an overall VNC_error_ of − 1.4 ± 6.1 HU. As per definition of VNC_error_, negative values indicate that the VNC attenuation values are lower compared to the expected attenuation of the base material; hence, that the iodine-associated attenuation has been slightly overestimated, a phenomenon known from earlier studies^10^. These findings are on the lower end of earlier reported VNC errors that range from − 1.2 to 15 HU^17,28,30^. This may be explained by the fact that in this study, unlike aforementioned investigations, a solid-state phantom was used. Here, the base materials exhibit attenuation different from water, whereas in most former studies aqueous dilutions of contrast media were used.

Interestingly, VNC_error_ was significantly lower in the medium-sized phantom as compared to small and large sizes. While the reason for this remains elusive, a possible hypothesis is that the medium sized phantom most closely resembles an average sized patient (350 × 250 mm) potentially effecting model assumptions in image reconstruction^2^. Other studies^28,30^ reported that smaller phantoms yielded higher accuracy of VNC images. In line with that, we found lowest standard deviation with small size phantom.

In accordance with earlier reports we found that VNC_error_ is independent from kernel setting and denoising level^9,27^. Regarding radiation dose, which was deemed to not significantly impact VNC_error_ based on our data, there are opposing reports^28,30^: While Van Hedent et al. report that lower radiation dose results in greater inaccuracy of VNC it needs to be acknowledge that their lowest dose (2 mGy) was markedly lower than the one used in this study (10 mGy)^28^. Similarly, Si-Mohammed et al. employing CTDI_vol_ of 2.5 mGy, 5 mGy and 10 mGy, reported lower accuracy of VNC images^30^. Of note, both studies used phantoms, which caused less attenuation compared to ours, enabling the use of such low doses.

The iodine content and the base material were deemed to significantly influence VNC_error_. In low concentrations, the iodine-associated attenuation is overestimated as indicated by a negative VNC_error_, while in concentrations greater than 3 mg/ml an underestimation was found. Similar observation have been made by different groups^4,29,30^, e.g. Hua et al. also report a shift from under- towards overestimation between iodine contents of 2.5 mg/ml and 5 mg/ml^4^. Opposed to this, Van Hedent et al. reported no impact of iodine concentration on VNC performance^28^; however, the lowest concentration they used was 2 mg/ml^28^. Regarding the base materials, data with attenuation different from water is sparse. A study by Kim et al. investigated the influence of different solvents on iodine quantification accuracy^11^. Interestingly they found an overestimation in amino acid based solutions for concentrations lower than 1 mg/ml, while an underestimation in higher concentrations^11^. They suggest that these differences might occur since in SDCT, two-material decomposition is performed for water and iodine. Hence, any additional material will tend to increase model uncertainty and therefore decrease performance^4,11^. This argumentation seems applicable to our findings as well. Further, it needs to be acknowledged, that a greater VNC_error_ can be expected in higher concentrations; however, we refrained from normalizing the error to ensure translation of our observations to clinical practice.

In general, our findings indicate a clinical applicability of VNC images as they show a reasonable accuracy under a variety of settings in an anthropomorphic phantom. Clinically, VNC reconstructions might be helpful in characterizing incidental findings, such as adenoma, or in increasing diagnostic confidence, e.g. in in hypodense lesions of the liver. For the latter, diagnosis is often found challenging in lesions < 1 cm; here, a lack of iodine uptake is highly suggestive of a cystic origin and may increase confidence.

Yet, there are limitations that need to be discussed. First, we did not test extreme values for radiation dose or phantom size but focused on scan parameters typically encountered in abdominal imaging and average patients. Second, we did not evaluate accuracy of iodine reconstruction as there are numerous earlier reports for SDCT and other DECT-approaches^8–11,27,28^. It would also be of interest to investigate the dependency of VNC_error_ on different iodine background concentrations for the parenchyma and other organs. Further, true iodine concentrations for the used phantom were unavailable as the specifications provided by the vendor were validated using a dual source CT, only. No subjective image analysis was performed on the visibility of lesions; as VNC and iodine maps are usually consulted to obtain quantitative information whereas other spectral results are better suited for this purpose^23,32,33^. The lesion size cut-off of 5 mm was chosen to ensure valid ROI placement. Assessment of actual performance for clinical decision making was out of scope of this study; hence, we do not advocate for the use of VNC instead of true non-contrast acquisitions if required a priori. Yet, our data together with earlier reports on clinical use-cases suggest that VNC closely resemble true non-contrast acquisitions irrespective of protocol settings and therefore may be used if deemed necessary after image acquisition. Another limitation is that we used a single CT scanner and interscan variation was not addressed in this study as those parameters were investigated in earlier studies (7).

In conclusion, the accuracy of virtual non-contrast reconstructions is independent from routinely applied dose ranges, kernel, and denoising level setting; however, we found a small dependency on patient size, base material, and iodine content.

## Supplementary information

Supplementary Tables.
