# Supplementary material for: Quantitative accuracy of virtual non-contrast images derived from spectral detector computed tomography: an abdominal phantom study
Source: Sci Rep. 2020 Dec 9;10:21575. doi: 10.1038/s41598-020-78518-5 (PMC7725817; doi:10.1038/s41598-020-78518-5)
Supplement: Supplementary file 1 — Supplementary Tables. [file 41598_2020_78518_MOESM1_ESM.docx]

***Quantitative Accuracy of virtual non-contrast images derived from Spectral Detector Computed Tomography: An abdominal phantom study***

*Jasmin A. Holz^1^, Hatem Alkadhi^2^, Kai R. Laukamp^1^, Simon Lennartz^1^, Carola Heneweer^1^, Michael Püsken^1^, Thorsten Persigehl^1^, David Maintz^1^, Nils Große Hokamp^1*^*

*Electronic Supplement 1*

**Mean and standard deviation of conventional, virtual non-contract, iodine, and VNC_error_ for all tissue types and regions of interest (ROI).**

|  | | **Conventional [HU]** | | **VNC [HU]** | | **Iodine [mg/ml]** | | **VNC_error_ [HU]** | |
| --- | --- | --- | --- | --- | --- | --- | --- | --- | --- |
| **Tissue type** | **ROI site** | **mean** | **SD** | **mean** | **SD** | **mean** | **SD** | **mean** | **SD** |
| Matrix | Matrix | 32.3 | 1.3 | 33.2 | 1.3 | 0.0 | 0.0 | -1.8 | 1.3 |
| Parenchyma | Spleen 1 | 96.7 | 1.4 | 58.9 | 0.7 | 1.4 | 0.0 | 4.9 | 0.7 |
|  | Spleen 2 | 96.3 | 1.7 | 58.9 | 0.9 | 1.4 | 0.0 | 4.9 | 0.9 |
| Type 1 | Lesion 6 | 48.5 | 2.3 | 42.5 | 3.5 | 0.2 | 0.2 | -2.5 | 3.5 |
|  | Lesion 7 | 47.2 | 3.5 | 39.4 | 3.6 | 0.2 | 0.1 | -5.6 | 3.6 |
|  | Lesion 9 | 46.0 | 3.1 | 39.5 | 3.0 | 0.2 | 0.1 | -5.5 | 3.0 |
|  | Lesion 12 | 44.9 | 2.7 | 38.7 | 4.3 | 0.2 | 0.2 | -6.3 | 4.3 |
| Type 2 | Lesion 1 | 59.9 | 4.2 | 46.1 | 4.2 | 0.5 | 0.2 | -7.9 | 4.2 |
|  | Lesion 5 | 57.5 | 2.9 | 49.4 | 2.9 | 0.2 | 0.1 | -4.6 | 2.9 |
| Type 3 | Lesion 2 | 41.8 | 3.2 | 28.6 | 3.3 | 0.5 | 0.1 | -6.4 | 3.3 |
|  | Lesion 3 | 45.5 | 5.9 | 30.6 | 5.7 | 0.6 | 0.2 | -4.4 | 5.7 |
|  | Lesion 14 | 44.0 | 4.3 | 32.5 | 5.9 | 0.5 | 0.2 | -2.5 | 5.9 |
| Type 4 | Lesion 10 | 120.5 | 4.1 | 55.5 | 4.0 | 2.4 | 0.2 | 1.5 | 4.0 |
|  | Lesion 13 | 115.2 | 3.8 | 48.4 | 2.9 | 2.6 | 0.1 | -5.6 | 2.9 |
|  | Lesion 15 | 114.5 | 4.4 | 51.2 | 3.3 | 2.6 | 0.2 | -2.8 | 3.3 |
|  | Lesion 17 | 118.9 | 4.8 | 51.5 | 7.8 | 2.7 | 0.4 | -2.5 | 7.8 |
| Type 5 | Lesion 4 | 182.4 | 5.6 | 59.1 | 4.4 | 4.9 | 0.2 | 5.1 | 4.4 |
|  | Lesion 8 | 184.0 | 5.6 | 56.4 | 2.5 | 5.0 | 0.2 | 2.4 | 2.5 |
|  | Lesion 11 | 180.2 | 6.0 | 59.1 | 5.7 | 4.8 | 0.3 | 5.1 | 5.7 |
|  | Lesion 16 | 180.7 | 5.4 | 61.8 | 5.2 | 4.5 | 0.3 | 7.8 | 5.2 |

*Electronic Supplement 2*

**VNC_error_ for different iodine content (none (0 mg/ml), low (0.3 & 0.4 mg/ml), medium (1.4 mg/ml), high (3.0 & 5.0 mg/ml)) dependence on scan and reconstruction parameters.**

| **size** | | | **S** | | | | | | **M** | | | | | | **L** | | | | |  |  |
| --- | --- | --- | --- | --- | --- | --- | --- | --- | --- | --- | --- | --- | --- | --- | --- | --- | --- | --- | --- | --- | --- |
| **Dose [mGy]** | | | **10 mGy** | | **15 mGy** | | **20 mGy** | | **10 mGy** | | **15 mGy** | | **20 mGy** | | **10 mGy** | | **15 mGy** | | **20 mGy** | |  |
|  | | | **mean** | **SD** | **mean** | **SD** | **mean** | **SD** | **mean** | **SD** | **mean** | **SD** | **mean** | **SD** | **mean** | **SD** | **mean** | **SD** | **mean** | **SD** | |
| **kernel** | **level** | **iodine cont.** |  |  |  |  |  |  |  |  |  |  |  |  |  |  |  |  |  |  | |
| Soft | none | none | -2.9 | 1.6 | -2.5 | 2.8 | -1.9 | 2.4 | -4.6 | 4.3 | -3.3 | 2.3 | -3.7 | 2.7 | -5.8 | 4.9 | -6.6 | 4.0 | -5.2 | 3.8 | |
|  |  | low | -3.6 | 4.0 | -5.0 | 4.7 | -6.8 | 4.0 | -3.1 | 4.9 | -5.9 | 6.9 | -8.6 | 4.6 | -6.5 | 2.9 | -0.1 | 7.0 | -6.6 | 2.1 | |
|  |  | medium | 6.4 | 0.2 | 5.2 | 0.0 | 5.1 | 0.3 | 4.3 | 0.8 | 4.4 | 0.7 | 4.1 | 1.5 | 4.8 | 1.4 | 4.3 | 0.5 | 4.6 | 0.9 | |
|  |  | high | -2.0 | 5.1 | 0.5 | 4.0 | 2.6 | 5.9 | 0.6 | 8.6 | -0.8 | 6.6 | -0.7 | 6.4 | 5.0 | 7.7 | 2.8 | 5.7 | 2.7 | 6.0 | |
|  | medium | none | -1.8 | 2.4 | -2.4 | 4.3 | -2.2 | 2.1 | -5.0 | 4.9 | -3.4 | 2.3 | -3.6 | 2.2 | -6.4 | 6.2 | -5.8 | 2.0 | -5.6 | 4.3 | |
|  |  | low | -2.8 | 4.9 | -4.9 | 4.2 | -6.5 | 4.0 | -2.8 | 4.4 | -5.9 | 6.5 | -8.1 | 4.7 | -6.1 | 3.0 | -0.4 | 8.1 | -8.0 | 3.5 | |
|  |  | medium | 6.2 | 1.0 | 5.1 | 0.3 | 4.7 | 0.1 | 4.0 | 0.4 | 4.6 | 0.6 | 4.2 | 1.1 | 4.2 | 1.1 | 4.4 | 0.1 | 4.9 | 0.5 | |
|  |  | high | -1.4 | 4.8 | 0.7 | 4.0 | 2.1 | 6.4 | 0.6 | 7.8 | -0.2 | 6.1 | -1.2 | 7.2 | 5.2 | 8.9 | 4.1 | 6.9 | 3.5 | 6.3 | |
|  | high | none | -2.9 | 1.6 | -2.5 | 4.0 | -2.6 | 2.0 | -5.0 | 4.4 | -3.6 | 1.9 | -3.1 | 3.0 | -5.2 | 4.6 | -7.1 | 3.4 | -5.3 | 3.5 | |
|  |  | low | -3.9 | 3.9 | -5.0 | 4.2 | -6.6 | 2.8 | -2.8 | 4.4 | -5.7 | 6.7 | -9.0 | 4.4 | -7.4 | 4.1 | -2.2 | 6.7 | -7.6 | 4.0 | |
|  |  | medium | 6.3 | 0.1 | 5.2 | 0.1 | 5.1 | 0.3 | 4.0 | 0.4 | 4.2 | 0.8 | 4.8 | 1.5 | 4.6 | 0.7 | 4.8 | 0.4 | 5.4 | 1.2 | |
|  |  | high | -0.6 | 4.1 | 0.7 | 4.2 | 2.0 | 6.2 | 1.1 | 8.8 | -0.1 | 6.7 | -0.6 | 7.2 | 4.7 | 8.4 | 4.0 | 6.8 | 3.5 | 5.6 | |
| Standard | none | none | -1.7 | 2.3 | -3.3 | 4.1 | -2.3 | 2.1 | -4.6 | 4.5 | -3.0 | 2.6 | -3.4 | 2.8 | -5.5 | 5.2 | -6.3 | 3.8 | -5.4 | 3.9 | |
|  |  | low | -2.6 | 5.8 | -4.6 | 3.7 | -7.0 | 3.5 | -3.6 | 2.2 | -6.0 | 6.2 | -7.5 | 3.2 | -6.4 | 4.4 | -1.5 | 7.5 | -5.3 | 2.1 | |
|  |  | medium | 6.2 | 0.8 | 4.7 | 0.8 | 4.9 | 0.4 | 4.8 | 0.1 | 4.3 | 1.1 | 4.9 | 1.6 | 5.1 | 0.5 | 4.2 | 0.7 | 5.5 | 0.6 | |
|  |  | high | -1.8 | 5.4 | 0.4 | 4.3 | 2.2 | 5.3 | -0.8 | 8.3 | -0.6 | 7.0 | -1.0 | 8.0 | 4.8 | 8.8 | 2.5 | 5.1 | 2.5 | 6.2 | |
|  | medium | none | -3.5 | 1.7 | -2.6 | 2.6 | -2.3 | 2.3 | -4.8 | 4.2 | -3.6 | 2.0 | -3.2 | 2.8 | -6.3 | 5.4 | -6.0 | 3.1 | -4.9 | 3.9 | |
|  |  | low | -2.6 | 5.7 | -4.6 | 4.2 | -6.9 | 3.6 | -3.7 | 4.3 | -5.1 | 6.7 | -8.4 | 5.2 | -6.5 | 3.7 | -1.3 | 7.2 | -7.3 | 3.8 | |
|  |  | medium | 6.4 | 0.3 | 5.2 | 0.1 | 5.0 | 0.1 | 4.5 | 0.5 | 4.3 | 1.0 | 4.4 | 1.0 | 5.0 | 0.8 | 4.3 | 0.6 | 5.5 | 0.9 | |
|  |  | high | -1.2 | 4.7 | 0.2 | 3.9 | 2.1 | 5.8 | 1.4 | 8.3 | -0.7 | 5.7 | -0.8 | 7.1 | 5.4 | 9.0 | 2.3 | 5.4 | 3.9 | 5.5 | |
|  | high | none | -1.9 | 2.4 | -3.0 | 3.7 | -1.6 | 1.5 | -5.1 | 4.6 | -3.2 | 2.1 | -3.1 | 3.2 | -5.5 | 4.6 | -7.1 | 4.2 | -4.5 | 2.9 | |
|  |  | low | -2.4 | 5.4 | -4.8 | 4.2 | -6.4 | 3.8 | -3.3 | 4.6 | -4.6 | 6.2 | -8.1 | 4.6 | -6.9 | 4.3 | -1.3 | 6.8 | -6.8 | 3.3 | |
|  |  | medium | 6.3 | 0.4 | 5.4 | 0.4 | 4.7 | 0.4 | 4.6 | 0.6 | 4.5 | 0.6 | 4.6 | 0.9 | 4.7 | 1.6 | 5.0 | 0.5 | 5.5 | 1.1 | |
|  |  | high | -0.1 | 4.0 | 0.5 | 4.0 | 2.3 | 5.9 | 1.3 | 8.1 | -0.0 | 6.3 | -0.3 | 6.7 | 4.6 | 9.2 | 3.4 | 6.9 | 3.5 | 5.8 | |
| Sharp | none | none | -1.6 | 2.1 | -3.2 | 4.0 | -1.4 | 1.7 | -4.3 | 4.4 | -2.7 | 1.4 | -3.9 | 3.0 | -6.8 | 4.9 | -5.2 | 2.5 | -5.6 | 3.6 | |
|  |  | low | -3.2 | 4.9 | -5.0 | 4.6 | -7.3 | 3.9 | -3.8 | 5.2 | -5.4 | 6.1 | -8.1 | 5.4 | -9.3 | 3.7 | -0.1 | 7.5 | -5.7 | 3.6 | |
|  |  | medium | 6.5 | 0.1 | 5.0 | 0.1 | 4.7 | 0.4 | 4.5 | 0.5 | 4.4 | 0.9 | 5.0 | 1.2 | 4.3 | 0.1 | 4.6 | 0.4 | 5.3 | 0.4 | |
|  |  | high | -1.1 | 5.0 | 0.0 | 4.1 | 2.8 | 6.4 | 0.8 | 7.7 | -0.5 | 6.9 | -1.2 | 7.8 | 4.5 | 8.0 | 4.0 | 7.9 | 3.2 | 6.2 | |
|  | medium | none | -2.7 | 1.7 | -2.9 | 3.9 | -2.0 | 2.3 | -4.7 | 4.5 | -3.7 | 2.4 | -3.1 | 3.3 | -5.2 | 4.3 | -5.4 | 2.5 | -4.7 | 4.8 | |
|  |  | low | -2.8 | 5.5 | -5.2 | 3.6 | -6.8 | 4.2 | -4.2 | 3.9 | -5.3 | 5.9 | -8.5 | 4.5 | -6.4 | 3.3 | -1.1 | 7.6 | -7.1 | 4.6 | |
|  |  | medium | 6.2 | 0.8 | 5.1 | 0.3 | 4.9 | 0.1 | 4.3 | 0.6 | 4.1 | 0.9 | 4.6 | 1.3 | 4.2 | 0.1 | 4.7 | 0.6 | 5.1 | 1.1 | |
|  |  | high | -1.6 | 4.9 | -0.0 | 3.8 | 2.3 | 6.0 | 0.6 | 8.2 | -0.3 | 6.7 | -1.1 | 7.7 | 5.3 | 8.0 | 2.3 | 6.0 | 4.0 | 4.9 | |
|  | high | none | -3.4 | 1.8 | -2.3 | 3.9 | -1.6 | 2.4 | -4.9 | 4.5 | -3.2 | 1.9 | -3.2 | 3.3 | -6.3 | 6.3 | -6.1 | 2.6 | -5.6 | 3.8 | |
|  |  | low | -3.0 | 4.6 | -5.1 | 4.2 | -7.2 | 3.8 | -3.1 | 5.4 | -5.9 | 5.9 | -5.2 | 3.5 | -6.7 | 3.2 | -1.4 | 7.1 | -7.6 | 1.8 | |
|  |  | medium | 6.1 | 0.1 | 5.3 | 0.2 | 4.9 | 0.2 | 4.6 | 0.1 | 4.3 | 0.8 | 4.6 | 0.9 | 4.9 | 0.8 | 4.9 | 0.4 | 5.2 | 0.8 | |
|  |  | high | -0.9 | 4.6 | 0.4 | 3.8 | 2.4 | 6.0 | 1.3 | 8.2 | -0.4 | 6.3 | -0.5 | 6.6 | 5.0 | 9.3 | 3.1 | 5.8 | 2.9 | 4.7 | |
